# Medication Reduction is Associated with Improved Activities of Daily Living and Muscle Strength in Post-Stroke Patients with Polypharmacy

**DOI:** 10.31662/jmaj.2025-0264

**Published:** 2025-11-21

**Authors:** Ayaka Matsumoto, Yoshihiro Yoshimura, Hidetaka Wakabayashi, Fumihiko Nagano, Sayuri Shimazu, Yoshifumi Kido, Takenori Hamada, Kouki Yoneda, Takahiro Bise, Aomi Kuzuhara, Ai Shiraishi

**Affiliations:** 1 Center for Sarcopenia and Malnutrition Research, Kumamoto Rehabilitation Hospital, Kumamoto, Japan; 2 Department of Rehabilitation Medicine, Tokyo Women’s Medical University Hospital, Tokyo, Japan; 3 Department of Rehabilitation, Kumamoto Rehabilitation Hospital, Kumamoto, Japan; 4 Independent Researcher, Kumamoto, Japan; 5 Department of Nutritional Management, Kumamoto Rehabilitation Hospital, Kumamoto, Japan

**Keywords:** polypharmacy, deprescription, activities of daily living, muscle strength, stroke rehabilitation

## Abstract

**Introduction:**

Polypharmacy is common among hospitalized post-stroke patients and is associated with adverse outcomes, such as decreased physical function and increased risk of drug-related complications. However, the association with functional outcomes, such as activities of daily living (ADLs) and muscle health remains unclear in this population. This study aimed to investigates whether reducing the number of medications during hospitalization is associated with improved ADL and muscle health in post-stroke patients.

**Methods:**

We conducted a retrospective observational study of post-stroke patients with polypharmacy (≥5 medications) undergoing inpatient rehabilitation. Patients were categorized based on whether the number of medications decreased during hospitalization. Outcomes at discharge included the motor domain of the functional independence measure (FIM), handgrip strength, and skeletal muscle mass index (SMI), assessed by bioelectrical impedance analysis. Propensity score (PS) matching and multivariate linear regression was performed to examine associations between medication reduction and each outcome, adjusting for clinically relevant confounders.

**Results:**

A total of 419 patients (mean age 75.9 years; 55.8% male) were included. Medication reduction occurred in 32.5% of patients, with a median decrease of two drugs. After PS matching, the cohort included 212 patients. In the multivariate analysis of this cohort, medication reduction was independently associated with higher FIM-motor scores (β = 0.105, p = 0.006) and greater handgrip strength (β = 0.073, p = 0.043), but were negatively associated with SMI (β = −0.158, p = 0.017).

**Conclusions:**

Medication reduction during hospitalization was associated with improved ADL and muscle strength, but were negatively associated with muscle mass, in post-stroke patients with polypharmacy. Medication optimization may support functional recovery in this population.

## Introduction

Polypharmacy is commonly found in older adults and is closely associated with adverse health outcomes. It has gained attention due to its link to higher risk of adverse drug events ^(1)^ and negative impact on health, such as cognitive decline ^(2)^, frailty ^(3), (4)^, and falls ^(5)^. Polypharmacy is also frequently observed in rehabilitation settings. In patients undergoing rehabilitation, polypharmacy has been associated with poorer prognosis, including decreased activities of daily living (ADL), cognitive function, nutritional status, and likelihood of discharge home ^(6), (7), (8)^. As a result, the issue of polypharmacy has gained growing attention in rehabilitation medicine. Among post-stroke patients, the prevalence of polypharmacy is particularly high, ranging from 33%-44% ^(9), (10)^, due to the frequent need for pharmacological treatment for secondary prevention and management of functional disorders. Stroke patients undergoing rehabilitation are especially vulnerable, as they commonly experience sarcopenia and functional decline during the subacute phase ^(11), (12)^. In this patient population, ADL and muscle health are key determinants of rehabilitation outcomes and long-term prognosis. Given the aging population and the increasing number of stroke survivors, optimizing pharmacotherapy―particularly through strategies aimed at reducing polypharmacy―during rehabilitation is becoming increasingly important to maximize functional recovery and prevent long-term disability.

However, there is a paucity of evidence regarding the impact of deprescribing on functional and skeletal muscle outcomes post-stroke. Although some studies have highlighted the potential benefits of medication reviews and interventions based on potentially inappropriate medications (PIMs) lists for patients with polypharmacy, the findings remain inconsistent. For instance, one study demonstrated that reducing PIMs, potentially prescribing omissions, and adverse drug reactions improved medication adherence in older patients ^(13)^. Conversely, another study found no significant difference in the incidence of geriatric syndromes, such as falls, cognitive decline, urinary incontinence, and pain, between patients receiving medication interventions and those receiving standard care ^(14)^. In rehabilitation settings, medication adjustments have shown the potential to reduce hyperpolypharmacy and optimize prescriptions, although no effect on cognitive function was observed ^(15)^. Additionally, in post-stroke patients with sarcopenia, reducing the number of medications during hospitalization was positively associated with improved nutritional intake ^(16)^. Despite these findings, there is limited evidence on whether deprescribing improves ADL and muscle health in stroke patients undergoing rehabilitation. If deprescribing for polypharmacy can be shown to enhance rehabilitation outcomes, it could lead to the development of more effective therapeutic strategies.

Therefore, the purpose of this study is to examine the association between medication reduction during hospitalization and ADL, muscle strength, and muscle mass at discharge in patients with polypharmacy after stroke.

## Materials and Methods

### Participants and setting

This cross-sectional study was conducted in a Japanese subacute care facility, targeting a 135-bed convalescent rehabilitation unit over a 4-year period from January 2020 to December 2023. The study targeted patients who had completed acute-phase management for cerebrovascular accidents at acute-care institutions, and were subsequently admitted to this subacute facility for rehabilitative care after clinical stabilization. To ensure a focused and appropriate patient cohort, several exclusion criteria were applied: refusal of participation, incomplete data sets, severe consciousness (Japan Coma Scale score of 3 ^(17)^), in-hospital mortality, transfer to other facilities or units for comorbidity management, and the use of fewer than five medications at admission (non-polypharmacy status) ^(1), (18)^.

The rehabilitation unit offered a comprehensive, individualized program tailored to each patient’s functional status and recovery goals. This program consisted of up to 3 hours of rehabilitative interventions daily, from admission through discharge, encompassing physical therapy, occupational therapy, and speech and language therapy. The rehabilitation regimen addressed multiple domains of functional recovery and ADL, incorporating interventions, such as limb-specific functional training, basic mobility exercises, ambulation practice, ADL training, and muscle strengthening. In addition to physical rehabilitation, the program included nutritional management. A registered dietitian provided specialized nutritional care, with emphasis on protein supplementation to promote muscular strength and recovery ^(19)^. This multidisciplinary, integrative approach, combining intensive rehabilitation with targeted nutritional support, was designed to enhance functional recovery in patients during the subacute phase following stroke.

### Data collection

Patient data, including demographic characteristics, anthropometric measurements, stroke classification, and the interval between stroke onset and hospital admission, were extracted from electronic health records. Comorbidities were quantified using the Charlson Comorbidity Index (CCI) ^(20)^, and pre-stroke functional status was assessed by a physician using the modified Rankin Scale (mRS) ^(21)^.

The functional independence measure (FIM) ^(22)^ was used to evaluate ADL, including both motor (FIM-motor) and cognitive (FIM-cognitive) domains. These assessments were performed collaboratively by nursing staff and rehabilitation specialists. Handgrip strength was measured by an occupational therapist using a Smedley hand dynamometer (TTM, Tokyo, Japan). The highest value from three trials of the non-dominant hand was recorded. For patients with hemiplegia, the unaffected hand was tested, while those with bilateral hemiplegia were assessed on both sides.

Muscle mass was evaluated by a physical therapist using bioelectrical impedance analysis (BIA) with a body composition analyzer (Inbody S10, Tokyo, Japan). Patients were examined in the supine position following at least 4 hours of fasting and 1 hour of physical rest. The skeletal muscle mass index (SMI) was calculated by dividing skeletal muscle mass by height squared. These assessments were completed within 72 hours of admission. The total number of rehabilitation therapy units provided during hospitalization was divided by the length of stay to calculate the average number of rehabilitation units per day. One unit was defined as 20 minutes of therapy.

Nutritional intake was assessed by nursing staff or a dietitian as the average percentage of food consumed during the first week of hospitalization.

### Medication management and group classification

A ward pharmacist led medication management for all patients throughout hospitalization. This included verification of prescribed medications at admission, weekly prescription reviews, patient education on proper medication use, assessment of adherence, and monitoring for adverse drug reactions. Pharmacotherapy potentially interfering with rehabilitation progress was reviewed and discussed collaboratively with attending physicians, nurses, and rehabilitation therapists in monthly multidisciplinary conferences, and adjustments were made when considered appropriate. While the initial proposal to adjust or discontinue a medication could be made by the attending physician based on daily clinical evaluation, all such decisions were subsequently reviewed by the ward pharmacist to ensure appropriateness. Furthermore, the patient’s response to any medication change, including effects on symptoms, clinical status, and rehabilitation progress, was continuously monitored by the multidisciplinary team. For example, in cases where psychotropic medications appeared to induce drowsiness and hinder rehabilitation, dose reduction or discontinuation was attempted through gradual tapering when clinically feasible ^(23), (24)^. Similarly, for medications suspected of contributing to anorexia or dysphagia and thereby exacerbating malnutrition, dose adjustments or discontinuation were considered as appropriate ^(25), (26)^. However, deprescribing was not guided by a formal protocol in this study.

The number of regularly prescribed medications at admission and discharge was assessed by the pharmacist based on medical records and prescription data. As-needed medications and topical formulations, such as eyedrops, patches, and inhalers were excluded from the count. Over-the-counter medications were not administered during hospitalization and thus were not included in the analysis ^(10)^. For the discharge medication count, only drugs that had been continuously prescribed and taken during the final week of hospitalization were included.

Patients were classified into two groups based on the change in medication number during hospitalization. Those with a reduction at discharge compared to admission were assigned to the medication reduction group, while those with an unchanged or increased count were categorized as the non-reduction group.

### Outcomes

The primary outcome measure was the FIM-motor score at discharge. The FIM comprises two distinct domains: the motor domain, encompassing 13 sub-items, and the cognitive domain, consisting of five sub-items ^(22)^. The motor domain evaluates performance across four key areas: self-care, sphincter control, transfer abilities, and locomotion. Each sub-item is assessed using a 7-point ordinal scale, ranging from complete dependence to full independence. The total FIM-motor score ranges from 13 to 91, with lower scores indicating greater functional dependence.

Secondary outcome measures included handgrip strength and SMI at discharge. These assessments were conducted within 72 hours before discharge and provided additional insights into patients’ physical function and body composition at the end of rehabilitation.

### Sample size calculation

To determine the required sample size, we used Power and Sample Size Calculation software, version 3.1.6, basing our calculations on findings from a previous investigation ^(16)^. That study reported a standard deviation (SD) of 26.2 for FIM-motor scores among hospitalized post-stroke patients. Assuming a true mean difference of 17 ^(27)^ between the medication reduction and non-reduction groups, our analysis indicated that a minimum of 38 participants in the reduction group and 76 in the non-reduction group would be necessary to reject the null hypothesis. This calculation was performed with a statistical power of 0.9 and an alpha error of 0.05, enhancing the robustness of our results.

### Statistical analysis

Results are presented as mean and SD for parametric data, median and interquartile range (IQR) for nonparametric data, and numerical percentages for categorical data. IBM SPSS Statistics, version 21.0 (IBM Corp., Armonk, NY, USA) was used for analysis. P < 0.05 was set for statistical significance. Bivariate analysis of baseline patient information and outcomes was performed by classification according to medication reduction. Comparisons between groups were made using t-tests, Mann-Whitney U tests, and chi-squared tests, depending on the type of data for the variables. The McNemar test was used to compare prescribing trends for drug categories at admission and discharge.

Given the retrospective cohort design of this study, patients were not randomized. Therefore, to adjust for potential indication bias regarding medication reduction, propensity score (PS) pair matching was performed. The selection of variables for the PS model was based on the authors’ clinical expertise and a review of previous literature, and included: age, sex, stroke type, days from onset to admission ^(28)^, history of stroke, premorbid mRS, CCI, FIM-motor at admission, FIM-cognitive at admission, handgrip strength, SMI, body mass index (BMI), Brunnstrom Recovery Stage (BRS) of the lower limb, energy intake, protein intake, and the number of medications at admission ^(6)^. To reduce confounding, two groups were then constructed using 1:1 nearest-neighbor PS matching with a caliper of 0.2 times the SD of the logit of the PS. This matching approach aimed to balance the distribution of observed covariates between the medication reduction and non-reduction groups. Subsequently, multiple linear regression analyses were performed on the matched cohort to evaluate the independent association between the degree of medication reduction and outcomes at discharge (FIM-motor, handgrip strength, and SMI). To adjust for potential confounders specific to each outcome, these models were adjusted for the respective baseline admission scores, with age and sex included as additional covariates. Multicollinearity was assessed using the variance inflation factor, with values below 10 considered indicative of acceptable collinearity.

### Ethics approval

This study adhered to the principles of the Declaration of Helsinki, and complied with ethical guidelines for medical and health research involving human subjects. The study protocol was approved by the Institutional Review Board of the participating hospital (approval number: 2022-17). Only data obtained during routine clinical care were used, with no additional procedures or sampling for research purposes. Due to the retrospective design, obtaining written informed consent was not feasible. However, an opt-out method was implemented in accordance with ethical standards, allowing individuals to withdraw at any time. This approach upheld patient autonomy while enabling the ethical conduct of retrospective research.

## Results

**Figure 1.**
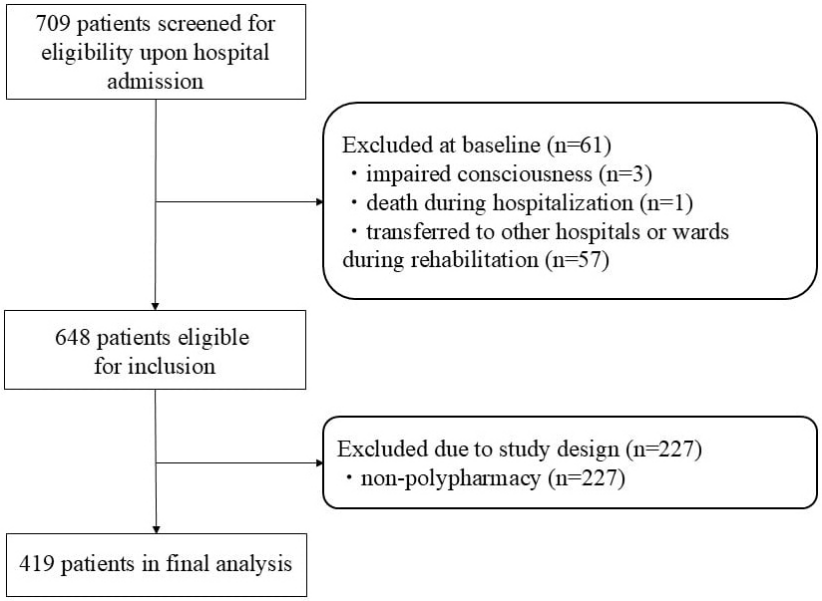
Flowchart of participant screening, inclusion criteria, and follow-up.

During the study period, 709 individuals with stroke were admitted to the facility. Following the application of exclusion criteria, which encompassed patients with impaired consciousness (n = 3), those who died during hospitalization (n = 1), and those transferred to alternative healthcare facilities or wards (n = 57), a cohort of 648 patients remained eligible for screening. From this group, 419 patients meeting the criteria for polypharmacy (defined as the concurrent use of ≥5 medications at admission) were ultimately included in the final analysis (Figure 1). Notably, polypharmacy was observed in 65.7% of the study population.

Table 1 delineates the baseline characteristics of the study cohort. The participants had a mean age of 75.9 years (SD 12.0), with men comprising 55.8% of the sample. At admission, the median medication count was 7 (IQR 6-9). Over the course of hospitalization (median duration: 84 days, IQR 53-129), the median change in medication count was 0 (IQR −2 to 1). A subset of 136 patients (32.5%) experienced a reduction in medication count by discharge, with a median decrease of two (IQR 1-3) medications. Inter-group comparisons revealed that the medication reduction group exhibited significantly lower rates of cerebral infarction, FIM-total, FIM-motor, FIM-cognitive, and BMI compared to the non-reduction group. Conversely, the reduction group demonstrated higher rates of cerebral hemorrhage and subarachnoid hemorrhage, superior upper limb BRS scores, and extended intervals between stroke onset and hospitalization. Among female participants, SMI was significantly lower in the reduction group, with a similar, albeit non-significant, trend observed in male participants. There were no significant differences between the two groups in energy intake, protein intake, or total rehabilitation units received during hospitalization. After PS matching, the two groups were well-balanced across all baseline characteristics (right side of Table 1).

**Table 1.** Baseline Characteristics of Participants and Group Comparisons by with and without Drug Decrease during Hospitalization.

	Crude population (N = 419)					PS-matched population (N = 212)				
	Overall	Medication reduction group	Non-reduction group	p value	SMD	Overall	Medication reduction group	Non-reduction group	p value	SMD
	(N = 419)	(N = 136)	(N = 283)			(N = 212)	(N = 106)	(N = 106)		
Age (year)	75.9 (12)	75.3 (13.4)	76.2 (11.3)	0.492	0.070	75.8 (12.9)	74.8 (13.7)	76.7 (11.9)	0.296	0.144
Sex, male	234 (55.8)	75 (55.1)	159 (56.2)	0.916	0.021	123 (58.0)	63 (59.4)	60 (56.6)	0.781	0.057
Stroke type				0.015	0.297				0.769	0.106
Cerebral infarction	301 (71.8)	86 (63.2)	215 (76.0)			143 (67.5)	69 (65.1)	74 (69.8)		
Cerebral hemorrhage	97 (23.2)	39 (28.7)	58 (20.5)			53 (25.0)	28 (26.4)	25 (23.6)		
Subarachnoid hemorrhage	21 (5.0)	11 (8.1)	10 (3.5)			16 (7.5)	9 (8.5)	7 (6.6)		
Onset-admission days	16 [12, 23]	18 [12, 28]	16 [12, 22]	0.033	0.271	17 [12, 25]	18 [12, 28]	16 [11, 22]	0.343	0.104
Premorbid mRS	1 [0, 2]	1 [0, 2]	1 [0, 2]	0.836	0.068	1 [0, 2]	1 [0, 2]	1 [0, 3]	0.271	0.122
Paralysis				0.578	0.147				0.658	0.176
Right	156 (37.3)	47 (34.6)	109 (38.5)			73 (34.4)	37 (34.9)	36 (34.0)		
Left	176 (42.0)	62 (45.6)	114 (40.3)			87 (41.0)	46 (43.4)	41 (38.7)		
CCI	3 [2, 4]	3 [1, 4]	3 [2, 4]	0.322	0.113	3 [1, 4]	3 [1, 4]	2 [1, 3]	0.346	0.116
Stroke history	117 (27.9)	37 (27.2)	80 (28.3)	0.907	0.024	59 (27.8)	33 (31.1)	26 (24.5)	0.358	0.148
BRS-finger	5 [2, 6]	5 [2, 6]	5 [3, 6]	0.092	0.200	5 [2, 6]	5 [2, 6]	5 [3, 6]	0.215	0.189
limb	5 [3, 6]	5 [3, 6]	5 [4, 6]	0.372	0.148	5 [4, 6]	5 [3, 6]	5 [4, 6]	0.471	0.136
upper	5 [3, 6]	5 [2, 6]	5 [3, 6]	0.031	0.241	5 [3, 6]	5 [2, 6]	5 [4, 6]	0.178	0.219
FIM (score)					0.241					
-Total	61 [33, 86]	50 [29, 81]	64 [36, 89]	0.004	0.312	60 [35, 82]	61 [34, 82]	59 [35, 83]	0.657	0.074
-Motor	43 [19, 62]	32 [15, 57]	46 [21, 67]	0.003	0.315	41 [21, 61]	41 [20, 61]	41 [21, 61]	0.485	0.108
-Cognitive	19 [10, 25]	17 [10, 24]	20 [11, 26]	0.017	0.246	18 [10, 24]	18 [12, 24]	17 [10, 23]	0.734	0.037
BMI (kg/m^2^)	22.4 [19.9, 24.7]	21.7 [19.3, 24.5]	22.6 [20.5, 24.9]	0.018	0.170	22.3 [19.8, 24.6]	22.2 [19.6, 24.9]	22.3 [20.0, 24.4]	0.745	0.147
Energy intake (kcal/kg/day)	26.8 [23.5, 30.2]	26.9 [23.4, 30.7]	26.8 [23.5, 30.1]	0.799	0.044	26.8 [23.6, 30.8]	26.5 [22.8, 30.8]	26.9 [24.4, 30.7]	0.151	0.254
Protein intake (g/kg/day)	1.0 [0.9, 1.2]	1.0 [0.9, 1.2]	1.0 [0.9, 1.2]	0.658	0.016	1.0 [0.9, 1.2]	1.0 [0.9, 1.2]	1.0 [0.9, 1.2]	0.325	0.206
HG										
-Men	23.8 [16.1, 31.3]	22.3 [14.2, 28.3]	25.4 [16.8, 33.0]	0.065	0.236	23.4 [17.1, 30.4]	24.0 [19.2, 32.1]	23 [16.55, 28.65]	0.365	0.155
-Women	11.5 [5.2, 17.3]	11.4 [0.0, 15.6]	11.9 [6.7, 17.6]	0.239	0.215	11.2 [3.0, 15.6]	10.0 [0.5, 15.4]	12.5 [5.2, 16.0]	0.364	0.150
SMI										
-Men	6.9 [6.2, 7.5]	6.7 [5.9, 7.3]	7.1 [6.7, 7.6]	0.059	0.245	6.8 [6.0, 7.4]	7.0 [6.1, 7.5]	6.8 [5.9, 7.4]	0.676	0.021
-Women	5.3 [4.7, 5.9]	5.2 [4.7, 5.6]	5.4 [4.9, 6.2]	0.030	0.427	5.2 [4.7, 5.7]	5.2 [4.8, 5.7]	5.2 [4.7, 5.6]	0.636	0.224
Rehabilitation^a^ (units/day)	8.2 [7.1, 8.6]	8.1 [7.0, 8.6]	8.3 [7.1, 8.6]	0.664	0.034	8.2 [7.1, 8.6]	8.2 [7.2, 8.6]	8.1 [7.0, 8.6]	0.231	0.111
LOS	84 [53, 129]	82 [53, 132]	85 [53, 123]	0.542	0.085	82 [51, 121]	76 [52, 113]	85 [50, 132]	0.518	0.081
Number of total drugs	7 [6, 9]	8 [6, 11]	6 [5, 8]	<0.001	0.672	7 [6, 9]	7 [6, 9]	7 [6, 9]	0.687	0.037
Decrease number	0 [-2, 1]	2 [1, 3]	-1 [-3, 0]	<0.001	2.381	1 [-1, 1]	1 [1, 2]	-1 [-2, -0]	<0.001	2.506

Data are expressed as means (standard deviation) for parametric data, while medians and 25th-75th percentiles (interquartile range [IQR]) were used to describe nonparametric data, and numbers (%) were used to describe categorical data. Comparisons between the two groups were made, depending on the type of variable data, using t-tests (two independent variables that were normally distributed), Mann-Whitney U tests (two independent variables that were not normally distributed), and chi-square tests (nominal variables). ^a^Rehabilitation therapy (including physical, occupational, and speech and swallowing therapy) performed during hospitalization (1 unit = 20 min). BMI: body mass index; BRS: Brunnstrom Recovery Stage; CCI: Charlson‘s Comorbidity Index; FIM: functional independence measure; HG: handgrip strength; LOS: length of hospital stays; mRS: modified Rankin Scale; SMD: standardized mean difference; SMI: skeletal muscle mass index.

Table 2 provides a comprehensive overview of prescribed medications at admission and discharge. At admission, the most frequently prescribed medications included proton pump inhibitors (PPIs), calcium channel blockers (CCBs), antiplatelet agents, statins, angiotensin II receptor blockers (ARBs), anticoagulants, laxatives, β-blockers, and dipeptidyl peptidase-4 inhibitors (DPP-4is). Upon discharge, the most commonly prescribed medications were CCBs, PPIs, laxatives, antiplatelet agents, statins, ARBs, anticoagulants, β-blockers, and DPP-4i. During hospitalization, the use of β-blockers, uric acid-lowering drug, single antiplatelet therapy, benzodiazepines, α-blockers for benign prostatic hyperplasia, and laxatives increased, whereas the use of dual antiplatelet therapy, diuretics, and PPIs decreased.

**Table 2.** Medications Prescribed at Admission and Discharge in the Medication Reduction and Non-Reduction Groups.

	On admission			At discharge			p Value
	Overall (N = 419)	Reduction group (N = 136)	Non-reduction group (N = 283)	Overall (N = 419)	Reduction group (N = 136)	Non-reduction group (N = 283)	
Antihypertensives							
Calcium channel blocker	249 (59.4)	83 (61)	166 (58.7)	249 (59.4)	69 (50.7)	180 (63.6)	0.999
Angiotensin II receptor blocker	181 (43.2)	61 (44.9)	120 (42.4)	180 (42.9)	48 (35.3)	132 (46.6)	0.999
angiotensin converting enzyme inhibitor	30 (7.2)	11 (8.1)	19 (6.7)	29 (6.9)	10 (7.4)	19 (6.7)	0.999
β-blocker	101 (24.1)	41 (30.1)	60 (21.2)	111 (26.5)	41 (30.1)	70 (24.8)	0.031
Diuretic	80 (19.1)	32 (23.5)	48 (17.0)	65 (15.5)	16 (11.8)	49 (17.3)	0.020
Lipid/ Uric acid Management							
Statin	203 (48.4)	59 (43.3)	144 (50.9)	196 (46.8)	54 (39.7)	142 (50.2)	0.265
Uric acid-lowering drug	44 (10.5)	13 (9.6)	31 (11.0)	63 (15.0)	14 (10.3)	49 (17.3)	0.001
Antithrombotic							
Antiplatelet drug (single)	151 (36.8)	33 (7.9)	118 (28.1)	187 (44.6)	51 (12.2)	136 (32.4)	<0.001
Antiplatelet drug (dual)	60 (14.4)	27 (6.4)	33 (7.9)	24 (5.7)	7 (1.7)	17 (4.1)	<0.001
Anticoagulant	120 (28.6)	35 (25.7)	85 (30)	122 (29.1)	38 (27.9)	84 (29.7)	0.791
Gastrointestinal Agents							
Proton pump inhibitor	300 (71.6)	100 (73.5)	200 (70.7)	248 (59.2)	58 (42.6)	190 (67.1)	<0.001
Ursodeoxycholic	24 (5.7)	12 (8.8)	12 (4.2)	33 (5.3)	4 (2.9)	18 (6.4)	0.824
Probiotics	46 (11.0)	20 (14.7)	26 (9.2)	40 (9.5)	10 (7.4)	30 (10.6)	0.441
Laxative	110 (26.3)	43 (31.6)	67 (23.7)	232 (55.4)	60 (44.1)	172 (60.8)	<0.001
Gastric mucosa protective drug	36 (8.6)	14 (10.3)	22 (7.8)	29 (6.9)	7 (5.1)	22 (7.8)	0.230
Antidiabetic Agents							
Dipeptidyl peptidase-4 inhibitor	98 (23.4)	27 (19.9)	71 (25.1)	104 (24.8)	26 (19.1)	78 (27.6)	0.210
Biguanide	43 (10.3)	13 (9.6)	30 (10.6)	50 (11.9)	11 (8.1)	39 (13.8)	0.118
Sulfonylurea	23 (5.5)	10 (7.4)	13 (4.6)	18 (4.3)	6 (4.4)	12 (4.2)	0.063
Sodium glucose co-transporter 2 inhibitor	17 (4.1)	4 (2.9)	13 (4.6)	19 (4.5)	2 (1.5)	17 (6.0)	0.727
Glinides	11 (2.6)	2 (1.5)	9 (3.2)	6 (1.4)	0 (0.0)	6 (2.1)	0.180
Glucagon-like peptide-1 receptor agonist	2 (0.5)	1 (0.7)	1 (0.4)	3 (0.7)	1 (0.7)	2 (0.7)	0.999
Psychotropics							
Melatonin receptor agonist	50 (11.9)	21 (15.4)	29 (10.2)	43 (10.3)	11 (8.1)	32 (11.3)	0.349
Orexin receptor antagonist	87 (20.8)	38 (27.9)	49 (17.3)	89 (21.2)	22 (16.2)	67 (23.7)	0.905
Benzodiazepines	24 (5.7)	12 (8.8)	12 (4.2)	59 (14.1)	15 (21.0)	44 (15.5)	<0.001
Antipsychotic drug	43 (10.3)	21 (15.4)	22 (7.8)	50 (11.9)	14 (10.3)	36 (12.7)	0.382
Antidepressant	53 (12.7)	20 (14.7)	33 (11.7)	63 (15.0)	9 (6.6)	54 (19.1)	0.212
Antidementia drug	25 (5.9)	12 (8.8)	13 (4.6)	26 (6.2)	9 (6.6)	17 (6.0)	0.999
Antiepileptic agent	31 (7.4)	11 (8.1)	20 (7.1)	40 (9.6)	12 (8.8)	28 (9.9)	0.093
Analgesics / Pain-related medications							
Non-steroidal anti-inflammatory drug	35 (8.4)	14 (10.3)	21 (7.4)	26 (6.2)	6 (4.4)	20 (7.1)	0.188
Acetaminophen	26 (6.2)	13 (9.6)	13 (4.6)	20 (4.8)	1 (0.7)	19 (6.7)	0.377
Neuropathic pain treatment	27 (6.4)	9 (6.6)	18 (6.4)	35 (8.4)	6 (4.4)	29 (10.2)	0.200
Urological Agents							
β_3_-adrenergic agonist	22 (5.3)	7 (5.1)	15 (5.3)	31 (7.4)	5 (3.7)	26 (9.2)	0.064
α_1_ blockers for benign prostatic hyperplasia	37 (8.8)	10 (7.4)	27 (9.5)	57 (13.6)	14 (10.3)	43 (15.2)	<0.001
Cholinergic agonist	23 (5.5)	12 (8.8)	11 (3.9)	31 (7.4)	7 (5.1)	24 (8.5)	0.169
Others							
Glucocorticoids	18 (4.3)	7 (5.1)	11 (3.9)	16 (3.8)	5 (3.7)	11 (3.9)
Expectorant	35 (8.4)	20 (14.7)	15 (5.3)	25 (6.0)	6 (4.4)	19 (6.7)	0.110
Activated vitamin D	39 (9.3)	15 (11.0)	22 (7.8)	38 (9.1)	12 (8.8)	23 (8.1)	0.999

Comparison of prescription rates of each medication at admission and discharge in the total patient population were made using McNemar tests.

Table 3 presents a comparative analysis of outcomes between the medication reduction and non-reduction groups in the PS-matched population. Univariate analysis revealed no statistically significant differences between these two cohorts in terms of FIM-motor scores, handgrip strength, or SMI.

**Table 3.** Univariate Analysis of Outcomes with and without Drug Decrease during Hospitalization in the PS-Matched Population.

	Overall	Medication reduction group	Non-reduction group	p Value
	(N = 212)	(N = 106)	(N = 106)	
FIM-motor	77 [47, 87]	77 [48, 87]	74 [47, 88]	0.689
HG				
-Men	25.5 [19.6, 32.2]	25.0 [20.3, 33.4]	26.3 [19.3, 31.7]	0.877
-Women	12.9 [6.8, 17.5]	12.1 [4.3, 15.8]	13.8 [9.3, 18.3]	0.254
SMI				
-Men	6.9 [6.2, 7.7]	6.9 [6.3, 7.7]	7.0 [6.2, 7.7]	0.894
-Women	5.4 [4.9, 5.9]	5.7 [4.9, 6.0]	5.1 [4.9, 5.9]	0.608

Data are expressed as medians and 25th-75th percentiles (interquartile range [IQR]) were used to describe nonparametric data. Comparisons between the two groups were made using Mann-Whitney U tests. FIM: functional independence measure; HG: handgrip strength; PS: propensity score; SMI: skeletal muscle mass index.

Table 4 presents the findings from the multivariate analysis in the PS-matched population, with no indication of multicollinearity among the variables examined. Multiple linear regression analysis revealed that a reduction in the number of medications during hospitalization was independently associated with improvements in FIM-motor at discharge (β = 0.105, p = 0.006) and handgrip strength at discharge (β = 0.073, p = 0.043). Conversely, a significant negative association was observed between deprescription and SMI (β = −0.158, p = 0.017).

**Table 4.** Multivariate Regression Analysis of Outcomes in the Population after Propensity Score Matching.

	FIM-motor at discharge				HG at discharge				SMI at discharge			
	β	B (95% CI)	p Value	VIF	β	B (95% CI)	p Value	VIF	β	B (95% CI)	p Value	VIF
Age	−0.255	−0.657 (−0.860 to −0.454)	<0.001	1.135	−0.193	−0.228 (−0.324, to −0.132)	<0.001	1.337	−0.318	−0.032 (−0.049 to −0.016)	<0.001	1.906
Sex (Men)	0.018	1.017 (−3.345 to 5.380)	0.646	1.093	0.079	2.032 (−0.098 to 4.161)	0.061	1.375	−0.015	−0.035 (−0.429 to 0.359)	0.857	1.932
FIM-motor on admission	0.731	0.842 (0.754-0.931)	<0.001	1.072	-	-	-	-	-	-	-	-
HG on admission	-	-	-	-	0.698	0.74 (0.643-0.837)	<0.001	1.693	-	-	-	-
SMI on admission	-	-	-	-	-	-	-	-	0.629	0.633 (0.420-0.847)	<0.001	3.169
Decrease drug number during hospitalization	0.105	1.162 (0.339-1.984)	0.006	1.005	0.073	0.369 (0.011-0.727)	0.043	1.006	−0.158	−0.071 (−0.129 to −0.013)	0.017	1.163

CI: confidence interval; FIM: functional independence measure; HG: handgrip strength; SMI: skeletal muscle mass index; VIF: variance inflation factor.

## Discussion

In this study, we examined the association between deprescribing and functional prognosis and muscle health in post-stroke patients with polypharmacy, and found two novel findings. First, reduction in the number of drugs during hospitalization was modestly associated with ADL at discharge in post-stroke patients undergoing convalescent rehabilitation. Second, a similar modest association was observed with muscle strength at discharge. Third, inverse association was found with muscle mass. Although the univariate analysis showed no significant difference between the groups for any of the outcomes, this is because in an unadjusted comparison, the effect of the intervention is masked by other powerful predictors. However, the finding of a significant association in the multivariate analysis, which adjusted for factors, such as baseline values, demonstrates that this statistical model was crucial for revealing the independent effect of medication reduction.

Reduction in the number of drugs during hospitalization was modestly associated with ADL at discharge in post-stroke patients with polypharmacy undergoing convalescent rehabilitation. While the associations observed were modest, they hold significant clinical relevance. In post-stroke patients, even small improvements in FIM-motor scores can translate to a reduced need for assistance with daily tasks, enhancing patient autonomy and reducing caregiver burden. This study corroborates the findings of several previous investigations on the effects of deprescribing in post-stroke patients ^(16), (29)^. However, prior studies were limited by small sample sizes, low precision, and a focus on patients with sarcopenia. Although generalizability remains limited due to the single-center design, our study included a larger and more diverse sample of post-stroke patients with polypharmacy. Polypharmacy has been consistently associated with adverse drug reactions and a decline in physical function ^(2), (30), (31)^. Moreover, an increase in the number of medications during hospitalization has been shown to negatively impact ADL at discharge ^(32)^. Reducing the number of prescribed medications may mitigate these risks and positively influence ADL improvement. Furthermore, in an age-stratified subgroup analysis, this positive association with ADL was particularly evident in the older adult group (≥65 years) and was not observed in the non-older group (Supplementary Table 1). This suggests that the benefits of medication optimization on functional independence may be greatest in older adults, who are more vulnerable to the adverse effects of polypharmacy. However, due to the small sample size of the non-older group and the possibility of insufficient statistical power, the absence of an effect in younger individuals could not be definitively ruled out. In this study, we did not statistically analyze the effects of individual drugs. However, when considering the results of this study in conjunction with the prescription changes shown in Table 2, for example, the use of antipsychotic drugs decreased in the drug reduction group, while it increased in the non-reduction group. Reducing the use of these drugs, as well as central nervous system-acting drugs, such as antidepressants and hypnotics, and drugs with high anticholinergic effects, may improve alertness, coordination, and balance, thereby directly improving ADL performance. Furthermore, the significant reduction in diuretics may have played a role in mitigating a major barrier to safe mobility in this vulnerable population by reducing the risk of orthostatic hypotension and dehydration. Conversely, some increases were also clinically justified; laxatives were essential for managing immobility-related constipation, a key aspect of ADL, while β-blockers were likely added for critical cardiovascular secondary prevention. The concurrent increase in benzodiazepines, likely to manage post-stroke anxiety or insomnia, underscores that the benefit is not derived from indiscriminately cutting medications. Instead, our findings suggest that the positive association with functional outcomes stems from a net reduction in the overall medication burden, achieved through a holistic optimization process that carefully weighs the risks and benefits of each drug. Such investigations would provide a more comprehensive understanding of the relationship between medication reduction and functional recovery in post-stroke patients. Further research is warranted to elucidate the impact of individual medications in greater detail.

Reduction in the number of drugs during hospitalization was modestly associated with muscle strength at discharge, but inverse association was found with muscle mass. To our knowledge, no prior studies have examined the impact of deprescribing on muscle health in this population. Recently, an association between polypharmacy and sarcopenia has been reported ^(33), (34)^. Although it was hypothesized that reducing polypharmacy would positively affect both muscle strength and muscle mass recovery, a key novel finding of our study is the dissociation of these effects. One possible reason for this is the difference in the way each drug affects muscle strength and muscle mass. Medications that influence muscle strength include antipsychotics ^(35), (36)^, anticholinergics ^(37), (38)^, and benzodiazepines ^(39)^. Benzodiazepine withdrawal reverses γ-aminobutyric acid type A-mediated central muscle relaxation and can raise handgrip strength. Likewise, the extrapyramidal symptoms and sedative effects resulting from the dopamine D2 receptor antagonism of antipsychotics are primary mechanisms contributing to muscle weakness. Anticholinergic effects are also linked to reduced handgrip strength, both through direct muscle relaxation by inhibiting acetylcholine and through cognitive impairment affecting attention and executive function. Therefore, deprescribing these drugs plausibly amplified the muscle-strength gains we observed. Patients with medication reduction during hospitalization experienced either a reduction in the use of these medications or no significant increase compared to those without deprescribing, which may have contributed to the recovery of muscle strength (shown in Table 2). However, this association with handgrip strength lost statistical significance in both the older and non-older subgroups in the age-stratified analysis (Supplementary Table 1). This is likely because the association observed in the full cohort was modest, and the statistical power was insufficient to detect this effect in the smaller subgroups. Therefore, the interpretation of the effect on muscle strength should be made with caution.

A key novel, yet unexpected, finding was the negative association between medication reduction and muscle mass at discharge. We first considered the influence of these medications known to influence muscle mass, such as glucocorticoids, sodium glucose co-transporter 2 inhibitors, glucagon-like peptide-1 receptor agonist, sulfonylureas, and glinides ^(40), (41)^. However, in the setting of this study, the frequency of prescription of these drugs was low, and there was little change in prescriptions from the time of hospitalization to the time of discharge in both the deprescribing group and the non-deprescribing group. We next examined the potential influence of hydration status, given the significant reduction in diuretics. Muscle mass in this study was measured using BIA, which is a non-invasive and convenient method, but its estimates are influenced by the body’s hydration status ^(42)^. However, there was no significant difference between the groups in the change in total body water during hospitalization (Supplementary Table 2), which could not explain the observed negative association. Therefore, the mechanism for this negative association remains unclear, and it is possible that unmeasured confounding factors, not limited to medications, may be at play. This unexpected result highlights the complexity of polypharmacy in the post-stroke population and underscores the need for further research in diverse populations to elucidate the specific biological pathways involved.

In this study, several baseline characteristics differed between the medication reduction and non-reduction groups. First, the reduction group had significantly lower FIM scores at admission and a longer interval from onset to admission, indicating a more extended stay in an acute-care facility. This may suggest that patients who required more extensive acute-phase treatment or had more severe functional impairment had initially been prescribed a larger number of medications for symptom management. Consequently, as their condition stabilized during rehabilitation, there was a greater potential for discontinuing unnecessary drugs. Second, this group had a higher proportion of hemorrhagic stroke and a lower proportion of cerebral infarction. Unlike patients with cerebral infarction who often require long-term secondary prevention therapies (e.g., antiplatelet agents, statins), patients with hemorrhagic stroke may have had acute-phase treatments (e.g., aggressive blood pressure management) tapered or discontinued during the subacute rehabilitation phase. This divergence in treatment pathways may have contributed to the greater opportunity for medication reduction. Therefore, the potential influence of these baseline characteristics on the likelihood of deprescribing and its subsequent association with functional outcomes must be considered when interpreting the results of this study.

Managing polypharmacy is crucial for enhancing ADL in stroke patients. While rehabilitation and nutritional management are well-established approaches for improving ADL and muscle strength ^(43), (44), (45)^, this study identified only a modest association between the reduction in the number of medications and improvements in these outcomes. Stroke patients experiencing polypharmacy often face challenges in improving ADL ^(9), (10)^; however, comprehensive medication management, including regular reviews, can optimize patient outcomes ^(46)^. Moreover, deprescribing has been shown to enhance nutritional intake ^(16)^, which may, in turn, amplify the benefits of nutritional interventions and potentially support improved rehabilitation outcomes. Ultimately, by improving physical function and fostering independence, thoughtful deprescribing may contribute directly to an enhanced quality of life for stroke survivors. Clinicians should identify high-risk patients who may be candidates for medication reduction therapy―such as those with polypharmacy, documented adverse effects like sedation, or a plateau in functional recovery―for targeted intervention, particularly at key care transition points. A multidisciplinary team approach, with pharmacists playing a central role in medication review, is essential for successfully implementing these strategies ^(46), (47)^.

This study has several limitations. First, due to its retrospective observational design, causal relationships between changes in medication number and rehabilitation outcomes cannot be determined. Second, as this was a single-center study, the generalizability of the findings may be limited. Third, although statistically significant associations were observed between medication reduction and some outcomes, the strength of these associations was weak, and their clinical relevance remains uncertain. Fourth, pharmaceutical care in this study was not provided according to a standardized protocol, and it is unclear whether consistent medication management was delivered across all patients. Fifth, the specific effects of individual drug classes on outcomes were not examined. In particular, information on dosage and duration of administration is important for considering the overall effect, and this information was also not considered. Sixth, muscle mass was measured using BIA, a method that is known to be influenced by the patient’s hydration status. In the future, a prospective multicenter randomized controlled trial is warranted to clarify the causal relationship between medication reduction and functional recovery after stroke. Stratifying patients based on medication classes and extending the follow-up period after discharge may help identify which medications contribute to improved outcomes and whether the gains in ADL and muscle strength are sustained, thereby strengthening causal inference.

In conclusion, the reduction in the number of medications was associated with improvement in ADL and muscle strength, but not muscle mass, in patients with polypharmacy after stroke. Screening for polypharmacy and deprescribing during hospitalization may contribute to improved rehabilitation outcomes. Our findings advocate for integrating polypharmacy screening and optimization into the standard care pathway for stroke rehabilitation.

## Article Information

### Acknowledgments

We would like to express our deepest gratitude to the Nutrition Support Team of Kumamoto Rehabilitation Hospital for their support of this study.

### Author Contributions

Conceptualization: Ayaka Matsumoto and Yoshihiro Yoshimura; Data curation: Ayaka Matsumoto^)^, Yoshihiro Yoshimura, Fumihiko Nagano, Sayuri Shimazu, Yoshifumi Kido, Takenori Hamada, Kouki Yoneda, Takahiro Bise, Aomi Kuzuhara, and Ai Shiraishi; Formal analysis: Ayaka Matsumoto and Yoshihiro Yoshimura; Investigation: Ayaka Matsumoto, Yoshihiro Yoshimura, Fumihiko Nagano, Sayuri Shimazu, Yoshifumi Kido, Takenori Hamada, Kouki Yoneda, Takahiro Bise, Aomi Kuzuhara, and Ai Shiraishi; Methodology: Ayaka Matsumoto and Yoshihiro Yoshimura; Resources: Ayaka Matsumoto, Yoshihiro Yoshimura, Fumihiko Nagano, Sayuri Shimazu, Yoshifumi Kido, Takenori Hamada, Kouki Yoneda, Takahiro Bise, Aomi Kuzuhara, and Ai Shiraishi; Supervision: Yoshihiro Yoshimura and Hidetaka Wakabayashi; Writing - original draft preparation: Ayaka Matsumoto, Yoshihiro Yoshimura and Hidetaka Wakabayashi; Writing - review and editing: Ayaka Matsumoto, Yoshihiro Yoshimura, Hidetaka Wakabayashi, Fumihiko Nagano, Sayuri Shimazu, Yoshifumi Kido, Takenori Hamada, Kouki Yoneda, Takahiro Bise, Aomi Kuzuhara, and Ai Shiraishi. All authors reviewed the results and approved the final version of the manuscript.

### Conflicts of Interest

None

### IRB Approval Code and Name of the Institution

Institutional Review Board of the Kumamoto Rehabilitation Hospital (approval no.: 2022-17).

## Supplement

Supplementary Material
