# Supplementary material for: Medication Reduction is Associated with Improved Activities of Daily Living and Muscle Strength in Post-Stroke Patients with Polypharmacy
Source: JMA J. 2025 Nov 21;9(1):198–208. doi: 10.31662/jmaj.2025-0264 (PMC12889877; doi:10.31662/jmaj.2025-0264)
Supplement: Supplementary Material [file 2433-3298-9-1-0198-s001.pdf]

Supplementary Table 1. Multivariate Regression Analysis of Outcomes in age-stratified Cohorts using Crude Population.

a) Older group (≥65 years) (N = 349)

	FIM-motor at discharge			HG at discharge			SMI at discharge		
	β	B (95% CI)	p Value	β	B (95% CI)	p Value	β	B (95% CI)	p Value
Age	−0.115	−0.415 (−0.635 to −0.195)	<0.001	−0.146	−0.22 (−0.308 to −0.132)	<0.001	−0.121	−0.019 (−0.031 to −0.006)	0.003
Sex (Men)	−0.074	−4.073 (−7.42 to −0.727)	0.017	0.099	2.253 (0.918-3.589)	0.001	0.187	0.423 (0.216-0.630)	<0.001
FIM-motor on admission	0.537	0.628 (0.526-0.73)	<0.001	0.012	0.006 (−0.035 to 0.047)	0.777	0.168	0.008 (0.003-0.014)	0.001
FIM-cognitive on admission	0.138	0.452 (0.170-0.735)	0.002	0.046	0.064 (−0.049 to 0.176)	0.269	−0.075	−0.012 (−0.027 to 0.004)	0.129
HG on admission	0.251	0.619 (0.416-0.822)	<0.001	0.718	0.738 (0.657-0.819)	<0.001	-	-	-
SMI on admission	-	-	-	-	-	-	0.684	0.623 (0.533-0.713)	<0.001
Decrease drug number during hospitalization	0.066	0.738 (0.140-1.336)	0.016	0.026	0.123 (−0.116 to 0.361)	0.313	−0.012	−0.006 (−0.044 to 0.032)	0.759

b) Non-older group (<65 years) (N = 70)

	FIM-motor at discharge			HG at discharge			SMI at discharge		
	β	B (95% CI)	p Value	β	B (95% CI)	p Value	β	B (95% CI)	p Value
Age	−0.070	−0.153 (−0.512 to 0.206)	0.397	−0.081	−0.128 (−0.398 to 0.142)	0.348	−0.005	−0.001 (−0.031 to 0.029)	0.960
Sex (Men)	0.051	1.979 (−6.603 to 10.561)	0.647	0.248	6.945 (0.487-13.402)	0.035	0.178	0.401 (−0.147 to 0.950)	0.145
FIM-motor on admission	0.387	0.269 (0.094-0.444)	0.003	0.010	0.005 (−0.127 to 0.137)	0.939	−0.108	−0.004 (−0.015 to 0.006)	0.394

FIM-cognitive on admission	0.252	0.55 (0.018-1.082)	0.043	0.093	0.146 (−0.254 to 0.547)	0.468	0.210	0.029 (−0.007 to 0.065)	0.114
HG on admission	0.290	0.423 (0.071-0.775)	0.019	0.533	0.562 (0.297-0.827)	<0.001	-	-	-
SMI on admission	-	-	-	-	-	-	0.772	0.782 (0.540-1.023)	<0.001
Decrease drug number during hospitalization	0.131	0.893 (−0.241 to 2.028)	0.121	0.052	0.258 (−0.595 to 1.112)	0.548	−0.048	−0.021 (−0.098 to 0.057)	0.589

FIM: Functional Independence Measure; HG: handgrip strength; SMI: skeletal muscle mass index.

Supplementary Table 2. Changes in Body Water Composition in the PS-matched population

	Overall	Medication reduction group	Non-reduction group	p Value
	(N = 212)	(N = 106)	(N = 106)	
Change in TBW (L)	0.40 [−0.57, 1.00]	0.45 [−0.30, 1.30]	0.15 [−0.67, 0.70]	0.774
Change in ECW/TBW	0.00 [0.00, 0.00]	0.00 [−0.01, 0.01]	0.00 [0.00, 0.00]	0.183

Data are expressed as medians and 25th to 75th percentiles (interquartile range (IQR)) were used to describe nonparametric data.

Comparisons between the two groups were made using Mann-Whitney U tests.

TB: total body water; ECW/TBW: extracellular water to total body water ratio.
